# Anti-nephrin antibodies in pediatric nephrotic syndrome: a new paradigm

**DOI:** 10.1007/s00467-026-07292-9

**Published:** 2026-04-23

**Authors:** Anne K. Mühlig, Felicitas E. Hengel, Jun Oh, Nicola M. Tomas, Tobias B. Huber

**Affiliations:** 1 Department of Pediatrics, University Medical Center Hamburg-Eppendorf, Hamburg, Germany; 2 III. Department of Medicine, University Medical Center Hamburg-Eppendorf, Martinistrasse 52, D-20246 Hamburg, Germany; 3 Hamburg Center for Kidney Health, University Medical Center Hamburg-Eppendorf, Hamburg, Germany

**Keywords:** Anti-nephrin autoantibodies, B-cell depletion, Relapse, Podocytes, Proteinuria, Biomarker, Minimal change disease

## Abstract

**Abstract:**

Circulating anti-nephrin antibodies have recently been identified as mediators in many cases of childhood nephrotic syndrome. After binding of the antibody to nephrin, a key component of the slit diaphragm, different intracellular signaling mechanisms lead to foot process effacement. These new insights into the pathophysiology of nephrotic syndrome may have the potential to influence clinical treatment strategies. First, the measurement provides a pathomechanism-specific serological biomarker of a previously clinically diagnosed renal disease. Second, it may have additional value for diagnosis and prognostication, as anti-nephrin antibodies are more common in steroid-sensitive forms and less frequently associated with multidrug-resistant forms. Furthermore, anti-nephrin autoantibodies have the potential to be used as biomarkers for disease activity and to estimate the risk of relapse before kidney transplantation, after conduction of appropriate clinical trials. Additionally, the detection of autoantibodies strengthens the role of B-cell targeting therapies and provides a potential rationale for further multicenter, prospective, randomized clinical trials using B-cell depletion to potentially reduce corticosteroid use and relapse rates in children. In the future, more targeted therapies such as the interference with downstream effects of antibody binding or the depletion of autoantigen-specific antibody producing cells might become possible. Finally, the detection of anti-nephrin autoantibodies provides an explanation for patients and their families with a previously elusive “idiopathic” disease in antibody positive patients. In summary, the identification of anti-nephrin antibodies in nephrotic syndrome now opens up new questions and paves the way for further experimental and clinical studies to improve diagnostic and therapeutic approaches.

**Graphical abstract:**

**Figure:**
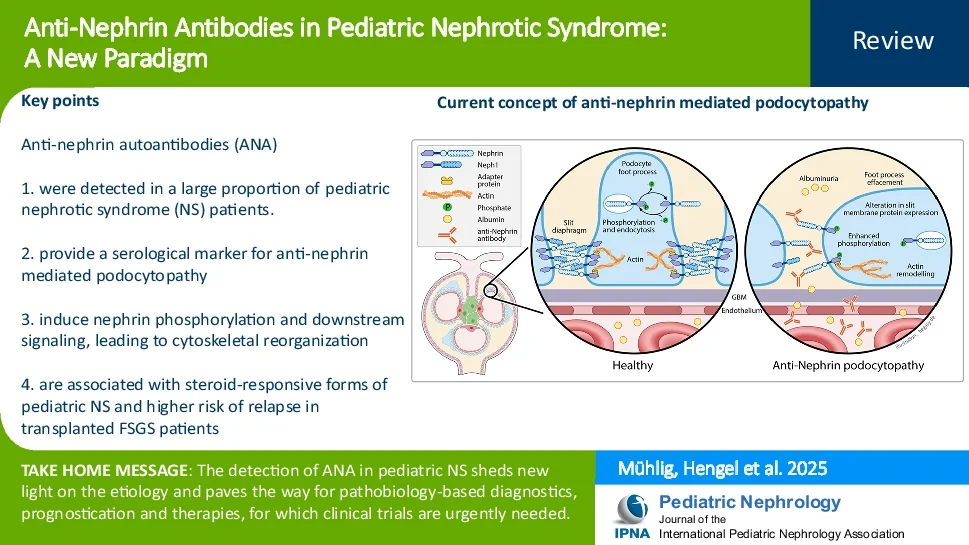
A higher resolution version of the Graphical abstract is available as Supplementary information

**Supplementary Information:**

The online version contains supplementary material available at https://doi.org/10.1007/s00467-026-07292-9.

## Introduction

Minimal change disease (MCD) and focal segmental glomerulosclerosis (FSGS), next to membranous nephropathy and sometimes IgA nephropathy, are found in biopsies from adult patients with nephrotic syndrome (NS) [1–3]. By contrast, if children show a good response to steroid treatment, pediatric nephrologists usually do not perform any kidney biopsy due to the potential risk for the patients. Studies have shown that MCD is the underlying histopathology in most steroid sensitive cases of childhood NS [4–7]. A renal biopsy does usually not provide any essential additional information, as the initial response to glucocorticosteroids is the best prognostic marker for the clinical long-term outcome so far [8]. Although glucocorticosteroids are rather effective in inducing remission, this unspecific treatment, without knowing exactly the underlying mechanisms, leaves an unsatisfying impression on the treating physicians.

Recent findings describing autoantibodies against nephrin, a protein of the glomerular slit diaphragm, in many children with NS shed some new light on the underlying mechanisms [9]. Even though there is a remaining proportion of anti-nephrin negative patients in this cohort, these findings could maybe pave the way for the development of new targeted therapies.

## Current understanding of childhood nephrotic syndrome pathogenesis

Childhood NS has been labeled “idiopathic” NS for a long time, as the underlying disease mechanisms have remained unclear. There is clinical and experimental evidence that the immune system is critically involved. Proteinuria, either in the initial presentation or in relapses, can occur after infections, e.g., of the upper respiratory tract, or different allergic reactions. Additionally, the response to glucocorticosteroids or other immunosuppressive drugs in the treatment of frequent relapsing forms [10] strengthens the hypothesis of an immune-mediated etiology. B-cell-depleting agents, e.g., rituximab (RTX), have shown efficacy in the maintenance of relapse-free intervals in patients with frequently relapsing forms of NS [11, 12] and have additionally highlighted the role of B cells in the development and course of the disease.

Although much research has been conducted on children with NS, the underlying proteinuria-inducing factor(s) remain elusive for many decades.

An autoimmune pathophysiology was suggested by many researchers because of the appearance of the first episode and the development of relapses after infections or allergic reactions.

The efficacy of B-cell-depleting therapies [11, 12] was another argument for autoantibodies being involved in NS development. On the other hand, the missing or only subtle IgG deposition in biopsies showing MCD were held as evidence of another—not antibody-mediated—immune-triggered mechanism. Although anti-nephrin autoantibodies have been identified in patients suffering from recurrent NS after transplantation in the context of congenital NS [13, 14], for many years researchers were not able to detect antibodies in acquired forms of NS in children or adults. Furthermore, besides B-cell-mediated theories, T-cell and cytokine involvement in pediatric NS has been in the focus of many researchers in the past. Several proposed mechanisms, including imbalance between Th2 and Th1 cells, a reduction in CD4 + T-cells, involvement of regulatory T-cells, and secretion of various factors have been proposed and have been nicely summarized before [15]. A paradigm shift in our understanding of childhood NS, at least for a sub-cohort of NS patients, was introduced by studies from Watts et al. [16] and our group [9], in which autoantibodies against nephrin were specifically detected in patients with MCD, FSGS, and children with immunosuppression-responsive forms of NS. Nephrin, a key slit diaphragm protein with extensive signaling function, is involved in the formation and maintenance of the slit diaphragm, and genetic nephrin mutations lead to severe congenital NS [17, 18].

## Association of anti-nephrin autoantibodies with nephrotic syndrome

Anti-nephrin autoantibodies in acquired forms of NS were described by Watts and colleagues in 2022, analyzing a cohort of adult and pediatric patients with MCD and FSGS [16]. Interestingly, the affected patients showed IgG co-localization with nephrin in their kidney biopsies [16].

Investigating large international patient cohorts, we detected anti-nephrin autoantibodies in approximately 44% of adult patients with MCD [9]. The percentage of positive patients rose up to nearly 70% of MCD patients in active nephrotic states without initiated immune suppression [9]. In children with “idiopathic” NS, the percentage was even higher and reached 90%, in patients with active disease and no immunosuppression at the time of sample collection [9]. The data from different studies also suggest higher prevalence in children (65% [19] to almost 90% [9]), compared to adults or mixed cohorts, where there have been detected prevalences between 29 and 70% [9, 16, 19]. Careful selection of patients seems to be essential, due to the fact that the antibody correlates with disease activity, which was also shown in most of the studies [9, 19, 20].

At study inclusion as well as across follow-up samples of remission and relapse, anti-nephrin antibody titers strongly correlated with disease activity [9]. In the light of their specificity for MCD, FSGS and childhood NS and their correlation with nephrotic disease activity, anti-nephrin antibodies could qualify as a new specific biomarker of primary podocytopathy. Today, it has not been shown in prospective trials so far whether anti-nephrin antibodies could predict the development of a relapse, nor should they influence any treatment decisions as multicentric randomized clinical trials are missing, but in some cases, the antibody titer could add another diagnostic tool to determine activity of the disease or help to identify correct diagnosis, especially in children or in adult patients with contraindications for a kidney biopsy. Furthermore, activity of the disease can be determined in anuric patients.

The measurement of anti-nephrin antibodies is challenging [21] and the prevalence varies between different studies. This could be explained by the fact that different methods for detection have been chosen. Different assays have been extensively tested and validated by Liu and colleagues [22]. Watts and colleagues used an indirect enzyme-linked immune-sorbent assay (ELISA), where antibodies in serum bind to nephrin, which was coated on plates and the bound antibodies were detected with secondary antibodies [16]. Unfortunately, ELISAs showed a high interindividual variation in background signals, thus we decided to use an immunoprecipitation in our studies. Here antibodies in serum are precipitated with recombinant nephrin, followed by Western blot detection of the eluated nephrin protein [9, 23, 24]. Especially in patients with low level anti-nephrin antibody titers [22] the immunoprecipitation, followed by Western blot, seems to be highly sensitive with an acceptable background positivity [22], although this method is quite time consuming. Nevertheless, this higher sensitivity explains the higher prevalence in our cohorts, compared to other studies. All the described difficulties and our own experience further highlight the reason why nephrin antibodies have been detected in transplanted patients suffering from nephrin mutations [13, 14], but not in non-genetic pediatric nephrotic syndrome, so far. The detection seems to be tricky probably because of relatively low titers of the antibody, which could only be detected with signal-enhancing or purification techniques [21].

For final validation of prevalence through different cohorts including different ages and ethnicities, standardized assays have to be established in order to test large cohorts and make the results comparable. Additionally, commercially available testing should be provided in the future to provide the possibility of testing to all treating centers.

## Potential diagnostical impact of anti-nephrin autoantibodies in pediatric NS

As children usually do not undergo a kidney biopsy, childhood NS is typically only assessed by clinical parameters such as edema as well as laboratory workup including serum albumin and proteinuria and the response to treatment. The detection of anti-nephrin autoantibodies and determination of anti-nephrin titers add additional information to the initial diagnostic procedure and follow-up analysis in patients, where we do not want to perform kidney biopsies [23]. Anti-nephrin autoantibodies seem to be associated with the development of NS in most of the children [9]. As the next logical step, such diagnostic potential and further implications need to be validated in clinical trials.

The presence of autoantibodies against nephrin is more common in patients with a histological diagnosis of MCD than FSGS [9]. Additionally, in children with NS responsive to steroid treatment, the prevalence of anti-nephrin is higher compared to steroid-resistant forms [24]. This was shown in our follow-up study, where 23 of 28 patients (82%), with active forms of steroid-sensitive NS, were found to be positive for anti-nephrin antibodies, compared to 13 out of 91 patients (14%) and 1 out of 46 patients (2%) with steroid-resistant non-genetic and genetic NS, respectively [24]. Anti-nephrin antibodies tend to correlate to a more severe NS phenotype, including higher proteinuria, lower serum albumin, and higher serum cholesterol [9, 19].

**Fig. 1:**
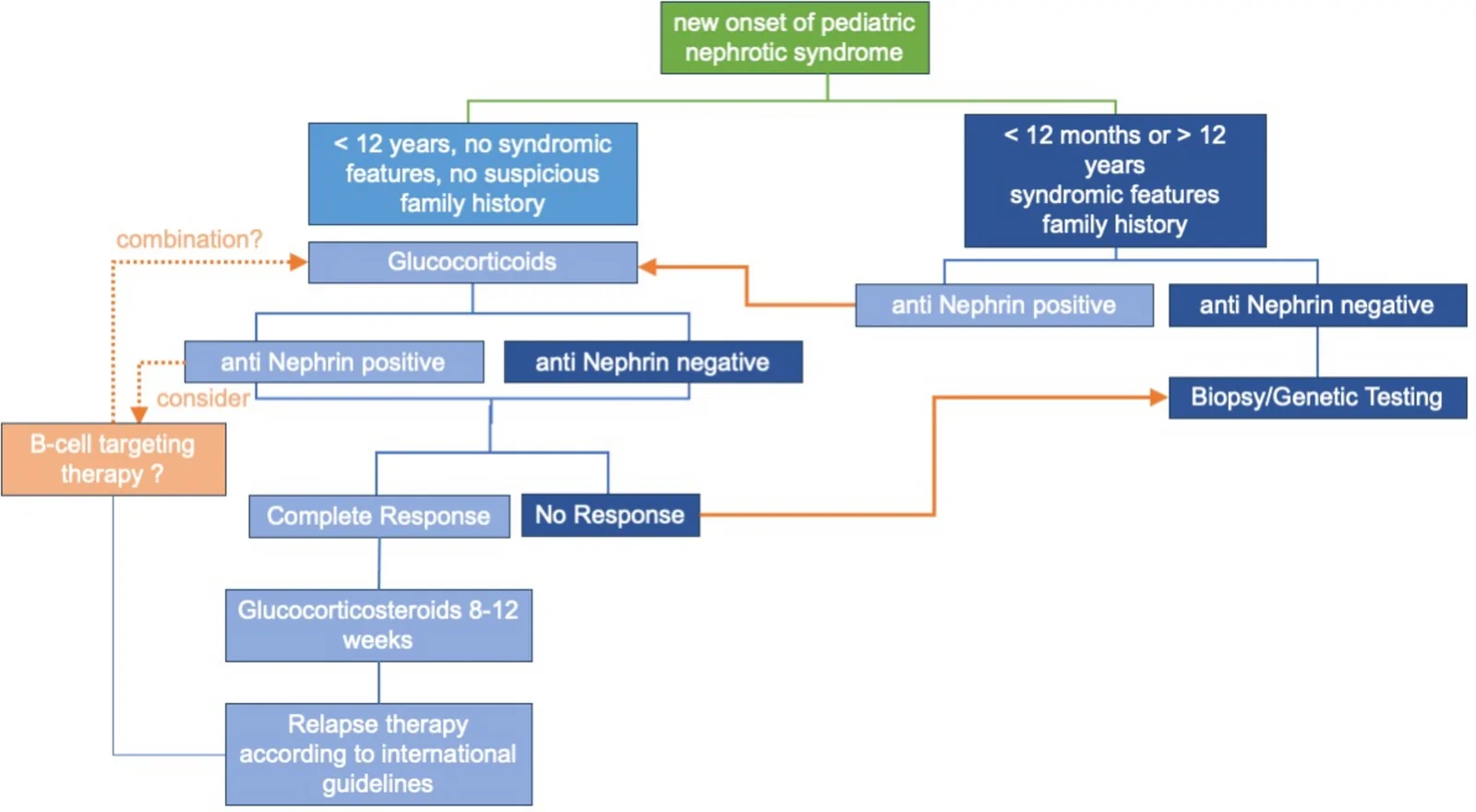
Potential diagnostical implications of anti-nephrin autoantibody measurement on stratification of pediatric nephrotic syndrome: red arrows are indicating a potential shift of “risk groups” of patients, depending upon the result of anti-nephrin antibody measurement. Start of prednisolone or further diagnostic procedures should not be postponed because of anti-nephrin measurement. Red dashed arrows are indicating potential alternative treatment strategies in anti-nephrin positive patients, given the new pathophysiological insights and in settings of prospective randomized controlled trials (the decision tree has been adapted from KDIGO and IPNA guidelines [46, 59]). In the current state anti-nephrin autoantibodies can be used in research and in future clinical trials, but should not yet be implemented into clinical decisions. Clinical trials have to show whether B-cell targeting therapies are justified in the initial treatment of NS. Because of safety concerns, rituximab should not be given in the early phases in children under 7 years of age [46]

As anti-nephrin antibody detection correlates with disease activity in adults and children [9, 16], the measurement of anti-nephrin antibodies could potentially add prognostic value to the diagnostic workup at the timepoint of initial diagnosis and has the potential to be used as a biomarker for disease activity [21], once the testing is standardized and available for all treating physicians and prospective clinical trials have been conducted. In the future, and after the respective clinical trials, it may help to select immunosuppressive treatment for the right patients and avoid ineffective treatment or unnecessary diagnostic procedures with severe side effects in patients with potentially non-immune-mediated and/or genetic NS. Figure 1 shows a suggestion of an investigational diagnostic and treatment algorithm; in case the urgently needed prospective randomized clinical trials will confirm the prognostic value and the potential of anti-nephrin antibody measurement as a biomarker for determination of disease activity.

## Meaning of anti-nephrin antibodies in post-transplant recurrence

Although the typical autoantibody-mediated NS in children rarely leads to kidney failure, there is a small fraction of patients with declining kidney function and the need for kidney transplantation. The measurement of autoantibodies in these patients could help to prognosticate recurrence and improve the outcome in transplantation. One study analyzed 14 pediatric patients with non-genetic forms of FSGS who had received a kidney transplant. 11 of those 14 patients suffered from recurrence and all 11 recurring patients were positive for autoantibodies against nephrin [25]. However, pre-transplant sera were not available for most patients, which could have indicated whether autoantibodies had been responsible for the initiation of FSGS and the recurrence in these cases. Furthermore, the rate of 100% antibody positivity in relapsing patients appears to be high and needs to be independently confirmed. Here, the choice of the nephrin protein used for immunoprecipitation could play an important role [22]. In another study, sera from patients being transplanted because of a podocytopathy were measured retrospectively. In this study pre-transplant circulating anti-nephrin autoantibodies were associated with a higher probability of recurrence: none of 17 antibody-negative patients showed recurrence in the transplant, whereas 8 out of 21 (38%) anti-nephrin positive patients relapsed [26]. Although further prospective and larger multicenter studies are needed, these results indicate that autoantibodies against nephrin and potentially other proteins of the slit diaphragm have a role in recurrent FSGS.

## Current concept of anti-nephrin-mediated podocytopathy

Nephrin is a transmembrane adhesion protein of 1241 amino acids, spanning the slit between interdigitating podocyte foot processes [17]. With its extracellular part, consisting of one fibronectin-like motif and eight IgG-like motifs, nephrin interacts with a multitude of binding partners through homo- and heterophilic interactions with each other, forming the porous structure of the slit diaphragm [27–30]. The cytoplasmic part of nephrin is involved in intracellular signaling and links the slit diaphragm to the actin cytoskeleton via numerous adaptor molecules [31], whose interplay is crucial for podocyte dynamics and function. An antibody binding its target antigen at such a crucial position and disturbing key signaling functions can be imagined to have severe pathological effects, as had been shown a long time ago by a single injection of a monoclonal anti-nephrin antibody leading to massive proteinuria in rats [32]. Furthermore, the specificity of anti-nephrin antibodies for primary podocytopathies and their strong correlation with disease activity fostered the hypothesis of a pathogenic role of anti-nephrin autoantibodies in the development of NS. To prove such pathogenic effect, animal models of active and passive immunization were employed and allowed further insight into anti-nephrin-mediated pathomechanisms [9, 33].

**Fig. 2:**
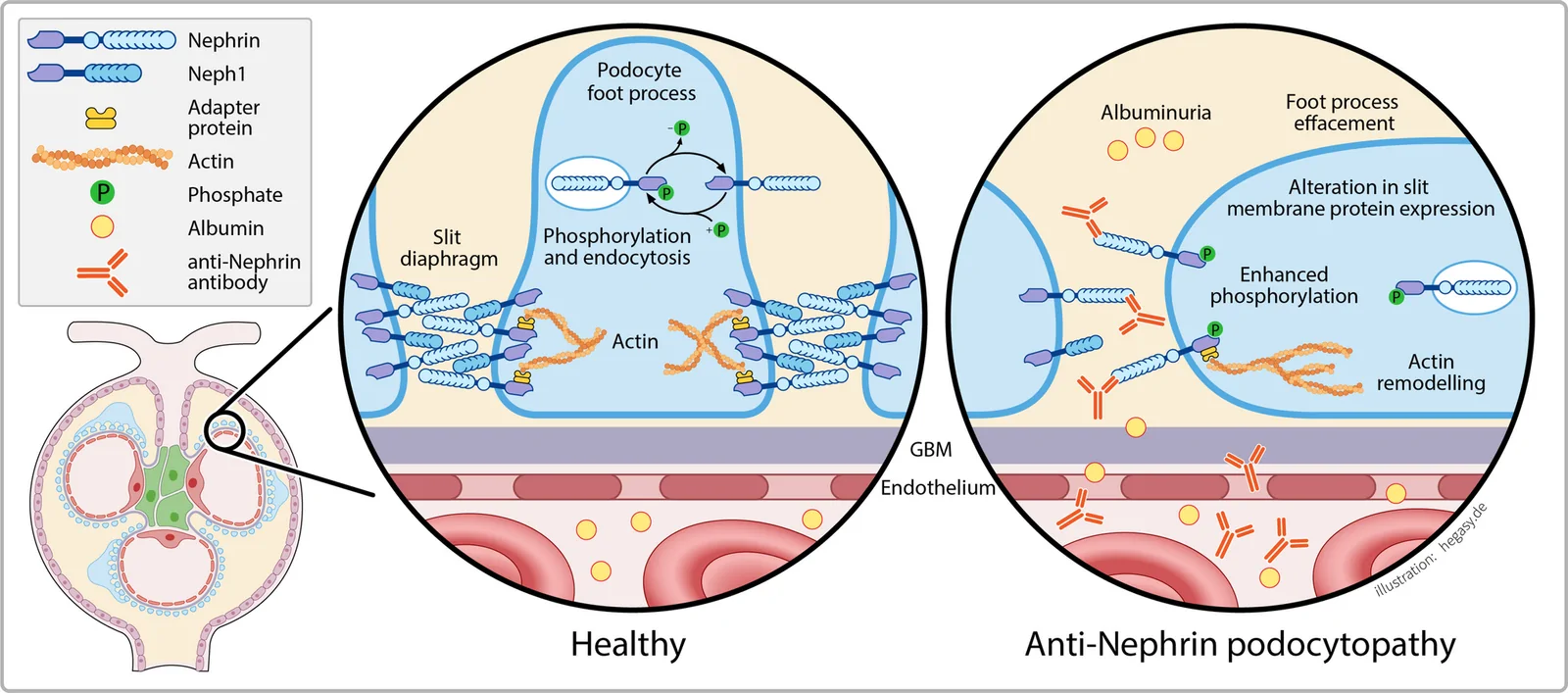
Current concept of development of autoimmune anti-nephrin antibody-mediated podocytopathy. Nephrin, an important protein at the podocyte slit diaphragm, interacts with Neph1 and nephrin molecules from neighboring foot processes to form the pores of the slit diaphragm. Nephrin phosphorylation is involved in endocytosis and turnover of nephrin molecules. Nephrin molecule phosphorylation and dephosphorylation is a precisely balanced homeostasis to maintain the slit diaphragm function. Disturbance of this balance, by binding of circulating anti-nephrin autoantibodies on extracellular domains of nephrin, leads to enhanced phosphorylation at group B tyrosines. This leads to enhanced endocytosis, actin polymerization, and changes in expression patterns of various slit diaphragm-associated and adhesion-associated proteins. This imbalance could end up in foot process effacement and podocyte loss in cases of FSGS

Mice that are immunized with the extracellular domain of nephrin develop anti-nephrin antibodies and nephrotic range proteinuria after 2–3 weeks, accompanied by a drop in serum albumin and an increase in cholesterol levels, indicating development of an NS in these animals [9]. In histology, there is no infiltration of immune cells, no complement activation, and no sclerosis, reflecting MCD in humans. Mouse IgG partially co-localizes with nephrin, detected by immunofluorescence staining, and electron microscopy shows foot process effacement with murine IgG being localized to remaining slit diaphragms [9]. Proteomic analysis of isolated glomeruli of nephrin-immunized mice revealed severe expression changes of several proteins associated with the slit diaphragm, podocyte adhesion, actin binding, and cytoskeleton. Additionally, a significantly increased phosphorylation of nephrin at tyrosin residue Y1191 was shown in autoantibody positive animals [9]. This phosphorylation site is known for its involvement in podocyte cytoskeleton reorganization and nephrin endocytosis [29, 34–38] (Fig. 2).

While these experiments demonstrate the development of a podocytopathy upon immunization with nephrin and thereby support an anti-nephrin-mediated, autoimmune pathogenesis, they do not demonstrate the direct pathogenicity of human anti-nephrin autoantibodies. In this direction, our group investigated autoantibody cross-reactivity with nephrin from several species. Anti-nephrin autoantibodies were derived from a patient with severe MCD who required plasmapheresis. These autoantibodies cross-reacted with rabbit nephrin, prompting us to transfer patient-derived purified IgG containing anti-nephrin IgG into rabbits. This experiment confirmed anti-nephrin autoantibodies were responsible for the development of podocyte foot process effacement and proteinuria in anti-nephrin-mediated podocytopathies [33]. Interestingly, anti-nephrin autoantibodies induced nephrin phosphorylation, but no significant complement activation or immune cell infiltration, mimicking the MCD phenotype that is seen in patients.

## Current concepts of alternative (anti-nephrin negative) mechanisms

Although anti-nephrin autoantibodies have been detected in a large proportion of patients suffering from pediatric NS in different studies, there are still cases which are anti-nephrin antibody negative. Other, so far unknown antigens, might additionally serve as targets for autoantibodies. This hypothesis is supported by IgG staining on kidney biopsies of patients with NS but no circulating anti-nephrin antibodies, which was identified in a sub-cohort of patients with NS (MCD or FSGS) [39], suggesting other proteins within the slit diaphragm to be targeted by antibodies. Conclusive with findings in anti-nephrin antibody positive patients [9, 19], these so-called anti-slit antibodies were associated with a better prognosis in kidney function survival and less glomerulosclerosis, and correlated to a more severe NS phenotype [39].

Additionally, Raglilanti et al. identified autoantibodies against Kirrel1 and podocin, both proteins which interact with nephrin within the slit diaphragm, in 2 different cohorts [40]. These antibodies appeared in smaller proportions (18% for podocin antibodies and 5% for Kirrel1 antibodies) of patients with NS [40]. Further studies are required to validate the existence, investigate their potential pathogenicity, and determine the influence on clinical course and prognosis of these antibody measurements. Unless this accounts only for the minority of the pediatric patients, identification of additional antigens and antibodies is important to decipher underlying disease mechanisms. In the past, there was a description of autoantibodies that targeted other podocyte proteins, e.g., UCHL-1 [41], CD40 [42, 43], or annexin A2 [44]. Nevertheless, the disease mechanism and underlying pathways have not been fully elucidated in these patients, and these antibodies have not been shown to be associated with disease severity. Large clinical studies, with standardized tests, to validate the prevalence and clinical impact of all antibodies should be done in the future.

As the prevalence of anti-nephrin autoantibodies is higher in children [9], additional antibodies will possibly become more important in adult patients. In adults, a more heterogeneous etiology and other pathogenic factors may play a role in the development of MCD and FSGS. Nevertheless, anti-nephrin negative cases should be analyzed further in detail to identify other antigens or non-antibody-mediated causes of the disease.

## Potential therapeutical implications of anti-nephrin antibodies

Until now, the therapy of childhood NS most likely does not directly target the causal underlying pathomechanisms [45]. IPNA 2022 recommends treatment with glucocorticosteroids (p.o. prednisolone or prednisone) in all pediatric cases of newly diagnosed nephrotic syndrome [46]. A biopsy and/or genetic testing should be considered if patients are younger than 12 months or older than 12 years of age and show extrarenal syndromic features, atypical features (e.g., acute kidney injury, hypertension, macroscopic hematuria, low C3 levels), or a suspicious family history [46]. More than 70% of pediatric patients with nephrotic syndrome (NS) show a very rapid and positive initial response to steroids.

The fact of this fast response to treatment with steroids is remarkable and is significantly different to what is seen in other antibody-mediated podocytopathies, such as membranous nephropathy (MN), where patients also show a slower and milder onset of the disease over weeks or months. Compared to MN, no immune deposits, which resolve slowly, occur in MCD or FSGS, which could on one hand explain the slower resolution of proteinuric disease in MN. Additionally, steroids themselves seem to have a direct effect in antibody-mediated disease and therefore further enhance rapid induction of remission. (1) Degradation/reduction of existing antibodies: we have shown in several cases that the anti-nephrin antibody levels in positive patients decline rapidly after initial treatment with prednisone [9, 23]. This measurement has not been shown in children yet, but the rapid induction of the remission suggests an even faster decline in pediatric patients. (2) Prevention of production of new antibodies through influence on and suppression of antibody producing cells. And (3) steroids seem to have a direct effect on podocytes, which has been shown in several studies: among other mechanisms, ligands of the glucocorticoid receptor seem to stabilize the cytoskeleton of podocytes by upregulating nephrin [47, 48] and steroids also seem to be involved in nephrin phosphorylation [49]. Therefore, the combination of missing huge antibody deposits in NS, the fast decline of a probably low titer antibody, prevention of further production and the additional podocyte stabilizing effect seem to be responsible for the remarkably fast induction of a remission in antibody-positive NS.

Aside from the quick initial response, most of the patients relapse in different frequencies. In frequently relapsing or steroid-dependent forms of NS, additional immunosuppressives, such as treatment with cyclophosphamide, mycophenolate mofetil, levamisole, calcineurin inhibitors, or rituximab (RTX) could be used as steroid-sparing agents. However, many of them relapse in the following years, and clinicians still lack reliable tools to predict the initial response to steroids, the frequency of future relapses, or the response to second-line immunosuppressants in cases of steroid dependence or resistance.

Treatment with RTX, an anti-CD20-antibody, plays an important role in the management of relapses in frequently relapsing or steroid-dependent forms of NS in adults [50] and children [51]. Patients show prolonged periods of remission (weeks to months) with RTX as a steroid-sparing agent, followed by a high probability of relapse once B-cells recover. This was seen in frequently relapsing or steroid-dependent NS, where RTX has been used as a second-line therapy or as a steroid-sparing agent. Nevertheless, given the new pathophysiological insights of antibody-mediated development of NS, targeting antibody-producing cells with drugs such as RTX could gain in significance in the treatment of NS, and prospective multicenter clinical trials are urgently needed.

Recently, a promising small study showed the efficacy of monotherapy with RTX to induce remission as a first-line medication in a first episode of the disease: 17 children were treated early in the course with 4 doses of RTX 375 mg/m^2^ [52]. 12 patients achieved complete remission, which was maintained for 78 weeks after B-cell depletion. Only one of the 12 responding patients relapsed after 85 weeks. The relapse rate in responding patients was lower than reported rates in pediatric NS patients treated with standard glucocorticoid therapy. The efficacy of RTX in the initial treatment of NS and the lower relapse rate have also been reported in a small cohort of adult patients [53] and in a prospective approach in anti-nephrin antibody positive patients by our group [9]. Although these studies involved only small cohorts and the data were analyzed retrospectively, they promise a new era of treatment that addresses the underlying cause of autoimmune-mediated podocytopathy. RTX seems to be safe for many children with NS; nevertheless, there are some known adverse effects and concerns about long-term safety, especially when RTX is administered in several cycles. The most common adverse events in children with NS treated with RTX are hypogammaglobulinemia in approximately 50%, clinically significant especially in younger patients, and infections or neutropenia in under 5% of the patients, which could lead to severe infections and even death [54, 55]. The use of RTX or other B-cell-depleting agents has been discussed in detail recently [56, 57]; nevertheless, without prospective randomized clinical trials, their use in first-line therapy is not justified yet. Furthermore, there is a small proportion of patients who do not relapse after an initial treatment course with steroids. In these cases, treatment with B-cell-targeting therapies should be avoided. Unfortunately, at the moment we do not have a good biomarker to recognize these benign courses beforehand. Therefore, also for these patients, multicentric prospective trials must be performed.

With initial studies suggesting an association of anti-nephrin antibodies with better response to therapy [9, 24, 39], the detection of anti-nephrin antibodies offers hope for improved prognostication of response to therapy. In addition, the advanced understanding of the underlying pathomechanisms allows the development of new treatment strategies based on targeting autoreactive antibody-producing cells. Future prospective randomized clinical trials analyzing whether anti-nephrin positive patients will benefit from specific B-cell targeting therapeutic approaches regarding relapse probability or frequency, or the development of chronification and development of FSGS, are eagerly awaited.

## Open questions

There is no doubt that the detection of anti-nephrin antibodies in childhood NS has changed the way pediatric nephrologists look at the pathophysiology of an idiopathic disease. Future studies may also modify the way these patients are managed, given the new insights into the pathophysiology of NS. Nevertheless, there are still open questions that should be addressed in future studies and experiments. The following part of this review discusses some of these open questions:

–Why do many children with NS stop relapsing when they reach puberty?

This remains one of the greatest mysteries in pediatric nephrology. While many patients relapse regularly during childhood, NS resolves when patients reach adolescence [58]. Many different reasons have been discussed in the past, including maturation of the immune system, influence of sex hormones, or increasing resistance of podocytes or other renal structures. The discovery of an autoimmune cause for most cases of childhood NS will shed a new light on this question. Therefore, multicenter prospective clinical trials need to consider the age, sex, and pubertal status of the children and adolescents enrolled, and follow them into adulthood. In addition to anti-nephrin measurements, clinical trials should also look at antibody titers and antibody-producing immune cells.

–Why is the outcome so different when the underlying pathophysiology seems so similar?

The outcome of NS varies from patient to patient. There are a few patients who never relapse after initial presentation and achieve complete remission after steroid treatment, while others develop steroid-dependent, frequently relapsing or even steroid-resistant and progressive forms. Initial retrospective studies have identified anti-nephrin positive forms of NS as steroid-sensitive with a lower risk of poor long-term outcome [24]. Further prospective studies need to confirm which patients are at higher risk for more complicated forms and whether there is a correlation with anti-nephrin antibody titers.

–What triggers the development of autoantibody-producing B cells? Why is the prevalence of NS higher in children than in adults?

Many cases of childhood NS are associated with the development of anti-nephrin antibodies [24] and the underlying histology then is usually MCD, whereas NS in adults is also caused by other glomerular diseases such as MN or IgA nephropathy, and the prevalence of anti-nephrin antibodies even in adults with MCD does not reach the prevalence in children [16, 24]. To explain the differences in different age groups, the reason for the development of autoantibody-producing B cells must be discovered. Regarding the age of the patients and the development of the first episode of treatment after minor infections or allergic reactions, the immaturity of the immune system in younger patients is suggested. Primary exposure to a specific antigen, such as a specific virus or certain bacteria, may trigger the immune system to produce antibodies against a self-antigen. Further basic research, in addition to the clinical trials mentioned, may shed more light on these questions.

## Summary and conclusions

In summary, recent studies suggest that autoantibodies against nephrin cause the majority of cases of childhood NS and there is reasonable hope that the term “idiopathic” could be replaced by more individualized and disease-specific terms, such as autoantibody or anti-nephrin antibody-mediated NS in a subgroup of patients in the future. While anti-nephrin autoantibodies currently represent an experimental biomarker primarily used in research and clinical trials rather than in routine clinical practice, future multicentric studies will be necessary to (1) determine the exact prevalence of anti-nephrin–associated podocytopathy across all age groups, ethnicities, and sexes, (2) monitor levels of antibody titers in response to therapy and during the follow-up of the disease and assess their role in prognostication of disease course and therapy response, (3) correlate findings with clinical parameters like time to relapse, the probability of relapses, and (4) in cases of transplants predict the risk of recurrence. These analyses will help in understanding the pathomechanisms of NS, which will be the basis for the development of more targeted new therapeutic approaches depending on the pathogenesis, by e.g., specific depletion of autoantibody producing cells or specific inhibition of signaling changes. Additionally, knowing the antigen, targeted epitope modifications might offer novel treatment options. For this, further research is needed to reach the next milestones in treatment of pediatric NS. Of course, there is still a long way to go to discover all underlying mechanisms, but the progress in disease understanding might replace the unsatisfactory term “idiopathic” with a more pathogenesis-based nomenclature, which is also important for patients and families who suffered from an “unknown” reason of their disease for such a long time.

## Supplementary Information

Below is the link to the electronic supplementary material.

Graphical abstract (PPTX 340 KB)

## Data Availability

No new data were created or analyzed in this study. Data sharing is not applicable to this article.
